# The small-for-size syndrome in living donor liver transplantation: current management

**DOI:** 10.1007/s13304-024-01964-7

**Published:** 2024-10-29

**Authors:** Luca Del Prete, Cristiano Quintini, Teresa Diago Uso

**Affiliations:** 1 General Surgery and Liver Transplant Unit, IRCCS Fondazione Ca’ Granda Ospedale Maggiore Policlinico di Milano, Milan, Italy; 2 Translational Medicine PhD Program, University of Milan, Milan, Italy; 3 Department of Liver Transplantation, Cleveland Clinic Abu Dhabi, Abu Dhabi, United Arab Emirates

**Keywords:** Hemodynamic, Graft inflow modulation, Small graft, Graft dysfunction, Portal hyperflow

## Abstract

Small-for-size syndrome poses a significant challenge in living donor liver transplantation, with potentially severe consequences including liver failure and death. This review explores the management strategies for SFSS, starting from the pathophysiology of the disease. SFSS arises from insufficient liver mass in the graft and hyperdynamic circulation in cirrhotic recipients, leading to portal hyperperfusion and subsequent liver injury. Risk factors include graft size, quality, recipient factors, and hemodynamic changes during transplantation.

Hemodynamic monitoring is crucial during living donor liver transplantation to optimize portal vein and hepatic artery flow. Prevention strategies focus on donor-recipient matching and intraoperative graft inflow modulation. Optimizing venous outflow and avoiding portal hyperperfusion is essential. Management of established small-for-size syndrome involves supportive care, pharmacologic interventions, and radiological and surgical options. Pharmacotherapy includes somatostatin analogues, beta-blockers, and vasopressin analogues to reduce portal flow and pressure. Surgical interventions aim to modulate portal flow and mitigate complications. Retransplantation may be necessary in severe cases, guided by persistent graft dysfunction despite liver flow modulations. In conclusion, preventing and managing small-for-size syndrome in living donor liver transplantation requires comprehensive assessment and tailored interventions. Advancements in graft/recipient matching, hemodynamic monitoring, pharmacologic and surgical techniques aiming to inflow modulation have improved outcomes, enabling successful transplantation even with ultra-small grafts.

## Introduction

In 1999, Kiuchi et al. [1] reported for the first time in living donor liver transplantation (LDLT) that the use of small-for-size grafts, defined as graft-to-recipient weight ratio (GRWR) of < 1%, is associated with a significant reduction in 1-year graft survival. The post-operative manifestation of this phenomenon was later defined as small-for-size-syndrome (SFSS), a clinical picture characterized by persistent hyperbilirubinemia, impaired hepatic synthetic function, encephalopathy, coagulopathy and refractory ascites [2–4]. In its most severe manifestation, SFSS results in liver failure, sepsis, and death.

This review describes the pathophysiology of SFSS, its risk factors, and strategies to mitigate its occurrence and severity.

## Pathophysiology and risk factors of SFSS

After transplantation, the insufficient liver mass of the graft, along with the hyperdynamic splanchnic circulation of the cirrhotic recipients, leads to an excessive portal vein flow (PVF). In the case of excessive portal hyperperfusion, the endothelial shear stress causes sinusoidal injury with erythrocyte zonal extravasation into periportal sinusoids, hepatocyte ballooning and cholestasis [5]. Furthermore, portal hyperflow causes decreased arterial flow due to a phenomenon known as the hepatic arterial buffer response [6, 7] (HABR), resulting in a higher risk of ischemic biliary injuries. Indeed, the relationship between PVF and hepatic artery flow (HAF) is regulated by HABR. In the perisinusoidal space of Disse, the potent vasodilator adenosine is produced at constant rates, but an increase in PVF causes an increase in the adenosine washout, resulting in profound arterial vasoconstriction. SFSS represents the extreme spectrum of an exaggerated HABR, where severe graft portal hyperperfusion causes severe hepatic artery vasoconstriction, resulting in arterial insufficiency, ischemia and poor graft survival [3, 5].

In its most severe forms, SFSS is associated with sepsis, which can be triggered by intestinal bacterial translocation due to increased mucosa permeability of the congested edematous bowel secondary to portal hyperflow[8].

Risk factors for SFSS can be divided into two main categories: unmodifiable, such as graft size, graft quality and recipient disease, and modifiable, such as graft inflow and outflow.

Extensive literature supports the notion that a GRWR < 0.8–1% or a graft/standard liver volume (SLV) ratio of < 40% is a risk factor for SFSS [9]. Although graft size is a known risk factor for SFSS, it is not the only one. Suboptimal grafts, for instance, are more prone to develop SFSS in cases of recipient-graft mismatch. Particularly, donor age > 45, donor body mass index (BMI) > 30, graft steatosis and long ischemia time contribute to SFSS development. Similarly, recipient functional reserve and disease severity as indicated by a model for end-stage liver disease (MELD) score > 20, Child class, and intensive care unit stay and dialysis at the time of transplant may contribute to SFSS [3, 9].

## Hemodynamic monitoring

The hepatic circulation is characterized by a complex network of blood vessels, receiving dual blood supply from both the portal vein and the hepatic artery. Normally, the hepatic artery contributes approximately 25% of the blood flow to the liver, delivering well-oxygenated blood at 30 mL/min per 100 g of liver weight and fulfilling 30–50% of the liver’s oxygen requirements. Conversely, the portal vein carries around 75% of the total blood flow to the liver, providing partially deoxygenated blood at a rate of 90 mL/min per 100 g of liver tissue, which meets approximately 50%-70% of the liver’s oxygen demands. During rest, the liver consumes about 20% of the body’s total oxygen.[10].

In the cirrhotic recipient and during liver transplantation, the interplay between portal vein, hepatic artery and portal shunts is crucial to achieve adequate pressure and flow into the new liver graft, avoiding SFSS. Therefore, the surgeon must consider liver hemodynamics during the different phases of liver transplantation when deciding whether or not to employ graft inflow modulation (GIM).

Historically, liver hemodynamics measurements have been classified into direct and indirect methods; the latter are less often used during liver transplantation given the availability of an open abdomen and exposed liver vessels. Flowmeters represent a diffused direct measurement method. These devices allow direct and continuous readings if applied directly around the vessel. One prevalent direct measurement technique involves transit time ultrasonic flowmeters, which emit ultrasound signals to measure blood flow directly around the vessel. Unlike Doppler flowmeters, transit time flow meters can accurately determine volumetric blood flow. However, challenges persist, including difficulty in simultaneously measuring portal vein and hepatic artery flow, technical issues related to vessel diameter changes, and the impact of factors like contact temperature, acoustic coupling medium, vessel probe fit, and probe diameter on readings. Moreover, surgical manipulation may induce arterial vasospasm, further complicating measurements.

In contrast, transabdominal ultrasound presents a non-invasive approach for assessing blood flow in the portal vein while preserving vessel integrity. Although measuring portal vein flows is comparatively simpler than assessing hepatic artery flows, both are essential for determining the total hepatic blood flow. This aggregate flow is derived by multiplying velocity by cross-sectional area, as determined through B-mode ultrasound. Nonetheless, fluctuations in the portal vein's diameter, potentially up to 40% during respiration, introduce the possibility of inaccuracies. Therefore, caution is warranted as absolute values may lack reliability [11].

## SFSS prevention

SFSS might be predicted and prevented by correct donor-recipient matching, intraoperative GIM and pre-existing portosystemic shunts management.

### Donor selection

Grafts from donors older than 45 years are associated with a higher risk of SFSS [12, 13]. Indeed, the donor age cutoffs for living donations are related to risks for SFSS development due to the decline in regenerative potential as age increases. In addition, older grafts are less tolerant of less portal vein pressure (PVP) > 15mmHg, resulting in a higher risk of GIM failure [14].

Acceptable graft steatosis for living donation depends on many factors. BMI alone is not an absolute criterion for graft steatosis [15]. Several authors considered 10% of graft macrosteatosis as a cut-off for living donation because of the relation between steatosis and increased release of inflammatory cytokines, lower potential for differentiation and regeneration of steatotic hepatocytes, and poor tolerance to ischemia–reperfusion injury [16, 17]. However, Bhangui et al. demonstrated that the safety threshold could be extended over 20% for young donors at low risk for small-for-size syndrome, without compromising graft function, donor safety and overall outcomes [18].

Guidelines from the 2023 Consensus Conference held by the International Liver Transplantation Society (ILTS), the International Living Donor Liver Transplantation Study Group (iLDLT), and The Liver Transplant Society of India (LTSI) recommend avoiding the use of grafts from donors older than 45 years or with more than 20% of macrosteatosis in combination with small-for-size grafts due to the higher risk of developing SFSS [19]

### Recipient selection

Recipient age and ABO incompatibility (ABOi) are not independent risk factors for SFSS [16, 20]. Abdallah et al. reported that a MELD score > 19 is associated with a higher risk SFSS [16, 21, 22], whereas another study set the threshold at MELD 26 [23].

Fulminant liver failure was shown to be less related to SFSS compared to chronic cirrhotic recipients because, in this setting, PVP may only be mildly increased. On the other hand, acute-on-chronic liver failure patients present with high PVP due to underlying cirrhosis and portal hypertension. Furthermore, in this patient category, the underlying inflammatory status is a known factor for SFSS [24]. These recipients have a higher metabolic requirement, therefore SFSS can be triggered if a small graft is transplanted [25].

### Outflow optimization

In a small graft for recipient size setting, optimizing the function of every transplanted graft is crucial. An unimpeded venous outflow plays a crucial role in portal vein hemodynamics and is associated with reduced graft regeneration, increased rate of SFSS and, ultimately, severe graft dysfunction. Different groups have reported the need to reconstruct the middle hepatic vein tributary (V5,V8). Wang et al. [26] reported that if the GRWR is > 0.86, the middle hepatic vein does not need to be reconstructed; however, if the GRWR is < 0.8, SFSS can be prevented when the middle hepatic vein is reconstructed. Other authors prefer reconstructing venous drainage when the size is significant (> 5 mm). Because the graft changes its position during intense regeneration (e.g., rotation), several groups have advocated for techniques that decrease the chance of venous obstruction in this setting. For right lobe transplantation, the slit technique has been proposed along with the use of a venous patch to augment the right hepatic vein outflow [27]. For left lobes, the septum between the left and middle hepatic veins is divided and two or three recipient hepatic veins are merged into a single cuff; the graft is then anastomosed onto the orifice [27]. Therefore, graft back table preparation of the venous outflow is a critical step in the prevention and mitigation of SFSS.

## Management

### General principle

The core of SFSS management is to reduce PVF and/or PVP whenever too high for the graft size, quality and recipient characteristics; this management strategy has been termed GIM.

The optimal timing for GIM in LDLT is still a matter of debate. It may be performed as an early GIM during liver transplantation to lower portal vein pressure and PVF to the graft, increasing HAF; or as a late GIM performed within the first 2 post-operative weeks. The latter is intended as a rescue procedure to improve graft function in the case of SFSS and to improve HAF [9].

The severity of the disease guides the management of established SFSS in LDLT after surgery. Medical care is recommended for pre-SFSS (grade A). Medical care, surgical or radiological inflow modulation (e.g., proximal splenic artery embolization—pSAE) is recommended in the portal hypertensive phase (grade B). Surgical or radiological inflow modulation and eventually liver retransplantation should be considered in the liver failure phase (grade C). SFSS severity grading determined by the ILTS-iLDLTG-LTSI Consensus Conference [29] is reported in Table 1.

**Table 1:** SFSS grading and management according to ILTS-iLDLTG-LTSI Consensus Conference

Grade	POD 7	POD 14	Graft Loss (%)	Treatment
A-pre-SFSS	T. Bil > 5 mg/dL	T. Bil > 5 mg/dL T. Bil > 5 mg/dL or ascites 1 L/d	< 9%	Supportive care, pharmacologic GIM
B-portal hypertensive phase	T. Bil > 10 mg/dL or INR > 1.6	T. Bil > 10 mg/dL and ascites 1 L/d	9–26%	Supportive care,, pharmacologic GIM, IR/surgical GIM
C-liver failure phase	T. Bil > 10 mg/dL and INR > 1.6	T. Bil > 20 mg/dL	59–77%	Supportive care,, pharmacologic GIM, IR/surgical GIM, possible liver retransplant

*GIM* graft inflow modulation; *INR* international normalized ratio; *IR* interventional radiology; *T.Bil* total bilirubin

### Supportive therapies

A recipient with SFSS is at higher risk of graft rejection, sepsis and fluid imbalance. The importance of supportive care for graft regeneration and recovery should not be underestimated. A small liver with a low GRWR may need lower immunosuppression due to its impaired metabolism [30–33]. Acute cellular mediated rejection would increase graft inflammation and worsen graft function and regeneration. Therefore, several authors recommend that adequate levels of immunosuppressive medications should be maintained in SFSS [29]. Although the recipient with SFSS is at a higher risk of sepsis, infectious disease prevention during SFSS is still under debate and there is no current agreement on whether or not to start antibiotic prophylaxis.

Fluid balance is one of the major challenges in SFSS. Although there is no strong evidence for a standardized practice, the management should focus on ascites management with diuretics, sodium restriction and paracentesis if needed. In recipients with high output loss through abdominal drains, serum sodium level should be monitored and administration of albumin 5% should be considered to avoid hypovolemia and consequent renal dysfunction [34].

For pain control, opioids should be used cautiously as a general rule because of the higher risk of adverse effects due to reduced hepatic metabolism in the setting of liver dysfunction and regeneration [35].

Extracorporeal support such as renal replacement therapy, plasmapheresis and adsorbent recirculating systems may help to reduce fluid overload and toxins, however there is no evidence that these approaches improve liver regeneration [36, 37].

### Pharmacological management

The core of the pharmacologic treatment of SFSS is to reduce PVF and PVP. Somatostatin is a peptide hormone that exerts its specific actions in the splanchnic vasculature. It inhibits stellate cell contraction, inducing sinusoidal dilation and reducing intraparenchymal resistance, resulting in a lower shear stress on microcirculation [38–40]. Furthermore, it reduces PVF by splanchnic vasoconstriction. Therefore, somatostatin is particularly valuable in mitigating the adverse consequences of hyperdynamic splanchnic and portal blood flow. Somatostatin produces a range of actions via binding to the five somatostatin receptor (SSTR) subtypes. However, owing to its brief half-life (1–2 min), continuous infusion is necessary. Somatostatin analogues, such as octreotide, exhibit heightened resistance to endogenous peptidases, leading to significantly prolonged half-lives compared to native somatostatin (approximately 2 h for octreotide). However, native somatostatin and its synthetic analogues have different affinities for the five SSTR subtypes: while somatostatin binds all five SSTR, octreotide binds SSTR 2, 3, and 5 and moderately to SSTR4, resulting in a lower effect compared to somatostatin.

In 2019, Troisi et al. [39] conducted a randomized control trial on LDLT recipients with portal hypertension managed with somatostatin 5ml bolus intraoperatively, followed by a 2.5 ml/h infusion for 5 days. The authors reported an intraoperative reduction compared to placebo of PVF and hepatic venous pressure gradient (HVPG) of 29.1% and 29.3%, respectively. Postoperatively, HVPG was lower in patients receiving somatostatin (81.7% Vs 58.8%; *p* = 0.0084), however no difference was observed in the PVF (*p* = 0.4185). Interestingly, somatostatin infusion counteracted the decrease in arterial flow (10% Vs 45%; *p* = 0.0431). Recently, the ILTS-iLDLT-LTSI Consensus Conference [29] recommended using somatostatin as an early intervention to decrease PVP (level of evidence moderate; strength of recommendation moderate).

As it is currently adopted in variceal bleeding prevention and as a treatment of portal hypertension in cirrhotics, β-blockers find a role also in SFSS management. Indeed, β_1_ blockers reduce portal hypertension, reducing cardiac output and β_2_ blockers induce splanchnic vasoconstriction [39–42].

Another pathway to reduce PVF is through vasopressin receptor activation by vasopressin or its synthetic analog terlipressin, which induces splanchnic vasoconstriction. In 2017, Reddy et al. [43] conducted a randomized clinical trial to evaluate the hemodynamic effects, clinical benefits, and safety of perioperative terlipressin infusion in adult LDLT. The authors did not demonstrate a reduction in post-reperfusion PVP, however recipients treated with terlipressin had less post-operative ascitic drain output and reduced renal injury. Other authors [44, 45] investigated the use of terlipressin started at the beginning of surgery and continued for two to three days. They reported a significantly decreased hepatic artery resistive index (HARI), and PVF and improved low systemic vascular resistance and mean arterial pressure, improved renal function and less ascites. Importantly, several authors had concerns about the use of terlipressin in daily practice because of its relation with higher intraoperative hyperlactatemia and symptomatic bradycardia; therefore, they recommended that the use of terlipressin be restricted to patients with high-volume ascites, and under close monitoring during drug infusion [43].

Prostaglandin E_1_ and prostacyclin induce vasodilation and help to reduce small graft congestion during SFSS, improving recovery from hyperammonemia and hyperbilirubinemia in a SFSS setting [46]. Suehito et al. [28] demonstrated a reduction of SFSS incidence from 25.4 to 3.4%, reduced ascites and lower serum bilirubin in LDLT recipients treated with prostaglandin E_1_ for two weeks after surgery. However, the use of prostaglandin during liver transplantation remains challenging due to its profound systemic vasodilator effects.

### Surgical management

When SFSS grade A progresses to grade B or C because of a failure of pharmacotherapy, surgical graft inflow modulation should be considered. Surgical management techniques are reported in Table 2.

**Table 2:** Interventional radiology and Surgical graft inflow modulation techniques

Invasive GIM type	When to consider	Pros	Cons
SAL/SAE[9, 62]	- PVF > 250 but < 500 mL/min/100g liver - PVP > 20 mmHg - HVPG > 15 mmHg	- Preserved spleen immunological functions	- Ineffective if PVF > 500 mL/min/100g - Temporary measure as collateral reforms with time
Splenectomy[52, 53, 55, 62]	- Intraoperative PVF > 500 mL/min/100g - Old donor age - Severe portal hypertension: - Graft-to-spleen ratio ≤ 70 g/mL - PVP > 15 mmHg after reperfusion - Splenic artery ligation failure	- Increase in post-operative platelet count - Removal of spleno-renal shunts - Removal of associated splenic artery aneurysm - Increase in hepatic serotonin which plays an important role in liver regeneration - Modulation of immunologic status in ABO-incompatible cases	- Higher intraoperative and post-operative bleeding requiring reoperation - Higher lethal infection incidence/sepsis, when compared to other techniques - High risk of PVT
Shunts[58, 64, 65, 73–75]	- SAL/splenectomy fails to reduce portal hyper-perfusion - PVF > 500 mL/min/100g or 3 times the expected flow	- High blood flow diversion - It can be used synergically with SAL/splenectomy (HPCS) - Almost 50% PVF and PVP reduction	- Risk of hepato-fugal flow requiring measurement to avoid hypoperfusion
Splenic devascularization[61]	- SAL fails to reduce portal hyper-perfusion - Splenectomy is considered high risk	- Profound reduction in hypersplenism effect - Reduces the harmful effects of splenectomy - Preserved spleen immunological functions	- Questionable efficacy in very small graft

*HVPG* hepatic venous pressure gradient;* HPCS* hemi−porto−caval shunt; *PVF* portal vein flow; *PVP* portal vein pressure; *SAL* splenic artery ligation

#### Splenic artery ligation (SAL) and proximal splenic artery embolization (pSAE)

SAL is performed intraoperatively, whereas pSAE is used as a postoperative salvage therapy. Both techniques aim to increase HAF and reduce portal hyperflow and are based on the concept that the level of spleen-derived perfusion is a crucial factor in portal inflow pressure [9, 47]. The relationship between SAL and PVP was initially investigated by Ito et al. [48],who recorded a statistically significant reduction in PVP after SAL in seven patients (16 mmHg Vs 11 mmHg; *p* = 0.01). These results have been confirmed by other authors [49]. Troisi et al. [9] recommend SAL as a first choice technique for GIM if the clamping test on the portal vein shows a significant increase in HAF and when a significant decrease in PVF and/or PVP is recorded by clamping the splenic artery.

pSAE can be performed during or as a savage therapy in the post-operative period. In 2007, Gruttadauria et al. reported six cases of pSAE with a decrease of bilirubinemia and ascites production; however one patient developed splenic abscess requiring surgery. In 2011, Quintini et al. [50] demonstrated a decrease in PV velocity after pSAE in six recipients with refractory ascites after deceased donor liver transplantation. Recently, D'Amico et al. [51] published a series of 30 recipients with refractory ascites (RA) or hydrothorax (RH) after LT, reporting a resolution RA or RH in 80% of the recipients within 3 months from pSAE. Considering vascular flow, six months after pSAE, significant reductions in mean PV velocity (78.1 ± 25.7 cm/s Vs 38 ± 11 cm/s, *p* = 0.001) and mean hepatic artery resistive index (0.8 ± 0.1 Vs 0.7 ± 0.1, *p* = 0.001) were reported, as well as a significant increase in mean hepatic artery peak velocity (44 ± 22 cm/sec Vs 90 ± 44 cm/s, *p* = 0.001). The procedure was complicated by two cases of splenic infarction, but with no cases of sepsis or splenic abscess. However, in this series only two LDLT recipients were included.

In addition to the intraoperative clamp test result, SAL or pSAE should be considered as first-line therapy when PVP is > 20 mm Hg and gradients between the portal vein and the hepatic veins are > 15 mm Hg, while PVF is between 250 and 500 mL/min/100 g liver.

The advantages associated with SAL or pSAE include their low morbidity profile and preservation of splenic immunological functions. Nonetheless, a notable drawback emerges when considering the efficacy of these procedures when PVF exceeds 500mL/min/100g of liver tissue. In this case, more complex techniques should be considered after careful intraoperative hemodynamics assessment.

#### Splenectomy

Splenectomy represents a more aggressive GIM technique that is more efficient than SAL or pSAE due to the interruption of all collateral vascularization supplying the spleen when the splenic artery flow is interrupted at its origin. Although it is the most common procedure for GIM in Asia, its recommendation is debated. While some studies suggest potential benefits, such as a notable 5 mmHg drop in PVP and enhancement in graft compliance, as evidenced by the ratio of PVF to PVP [14, 52], according to the Kyushu Group, other authors have raised questions regarding its overall advantages.

Use of splenectomy for GIM is restricted to carefully selected LDLT-recipients. These patients typically exhibit specific characteristics, including an intraoperative PVF > 500 mL/min/100g, advanced donor age surpassing 45 years, severe portal hypertension indicated by a graft-to-spleen ratio of ≤ 70 g/mL, PVP > 15–20 mmHg following reperfusion [14, 53, 54], or failure of a previous SAL.

Aside from representing a valid GIM strategy, other potential benefits associated with splenectomy include the removal of spleno-renal shunts, augmentation of post-operative platelet count and removal of splenic artery aneurysm if present. Moreover, the increase in hepatic serotonin levels post-splenectomy is recognized for its significant role in liver regeneration [14, 53, 55, 56]. In addition, splenectomy is posited to modulate the immunologic status in ABO-incompatible cases, and historically, it was purported to enhance tolerance to pegylated interferon and ribavirin in hepatitis C virus cases, albeit this application is now considered obsolete.

In 2014, colleagues from Taiwan published the Taipei Algorithm for GIM, which considers not only PVP, but also PVF and HAF. Feng AC et al. promotes splenectomy if, in the absence of outflow obstruction and technical anastomosis mistakes, PVF is ≥ 250mL/min/g, PVP is ≥ 20mmHg and HVPG is ≥ 15mmHg; or if PVF is ≥ 250mL/min/g and PVP is 15-20mmHg but HAF is < 100mL/min or PVF is 100-250mL/min/g and HAF is < 100mL/min [53].

Nevertheless, the procedure is not without its drawbacks. Splenectomy is linked to higher rates of intraoperative and post-operative bleeding (10–14%). Moreover, sepsis rates of 7%-49% and pancreatic fistula rates of 3.1%-23% have been reported. In addition, the risk of portal vein thrombosis is 1.7%-5.7% following splenectomy [14, 55, 57–62]. Importantly, the anatomy of the inferior mesenteric vein is critical for post-operative management of anticoagulants and anti-aggregant therapy. According to our experience, if the inferior mesenteric vein arises from the splenic vein, acetylsalicylic acid should be administered for six months, whereas if the inferior mesenteric vein arises from the superior mesenteric vein, anticoagulation should be added to anti-platelet therapy for three months.

The ILTS-iLDLT-LTSI Consensus Conference [29] document recommends SAL or splenectomy as a surgical treatment choice in post-transplant Grade B SFSS cases that fail pSAE (Level of evidence: low: strength of recommendation: moderate).

#### Splenic devascularization

Originating from the innovative efforts of the Asan team in Korea and introduced into clinical practice since 2013 [61], splenic devascularization represents an evolution of SAL. This approach involves an aggressive form of arterial ligation aimed at significantly reducing the hypersplenism effect, a common complication encountered in liver transplantation. Unlike splenectomy, which involves complete spleen removal and carries inherent risks, splenic devascularization selectively targets arterial supplies to the spleen while leaving the splenic vein and the spleen itself untouched. This strategy mitigates the harmful effects associated with splenectomy and preserves the spleen's essential functions. However, despite its promising outcomes, particularly in alleviating SFSS-related complications, the efficacy of splenic devascularization may be limited in cases involving very small grafts. Nonetheless, the technique'’s precise methodology, which involves ligating all arterial supplies to the spleen except for small branches originating from the pancreas (division of gastrosplenic ligament, ligation of right gastroepiploic artery, ligation of splenic artery), coupled with post-operative antibiotic management for seven days, underscores its potential as a valuable tool in optimizing outcomes in LDLT. Further research is warranted to elucidate the optimal application of splenic devascularization, particularly in scenarios involving very small grafts, to enhance its effectiveness and refine its clinical utility.

#### Surgical shunts

The creation of surgical porto-systemic shunts provides an alternative route for blood to bypass the liver, alleviating the portal hyperflow on the graft and mitigating the risk of SFSS-related complications. Shunt formation contributes to optimize graft function and enhance post-transplant outcomes by redirecting excessive PVF away from the liver.

Shunt formation achieves significant reductions in PVF by 30–65% and a notable drop in PVP by 8–10 mmHg. However, many authors consider shunt creation as a secondary surgical procedure, utilized only when SAL, pSAE or splenectomy fail to reduce portal hyperperfusion sufficiently [9, 63, 64].

Despite its efficacy, shunt formation presents several drawbacks. Even with meticulous calibration, shunts may result in uncontrolled shunting, potentially leading to complications. Various types of portosystemic shunts exist, each with its implications. For instance, the hemiportocaval shunt, often used for left lobe grafts, may synergize with SAL/splenectomy but carries the risk of substantial PVF and PVP reduction, up to nearly 50%. Similarly, portocaval and mesocaval shunts entail high blood flow diversion, necessitating careful monitoring to avoid hepato-fugal flow and subsequent hypoperfusion. In addition, shunts like the meso-renal shunt, mesocaval shunt plus mesenteric disconnection, and splenorenal shunt offer alternatives, each with unique considerations regarding efficacy and risk profiles.

Yamada et al. [64] reported in the Kyoto algorithm for left lobe live transplantation the use of hemi-portocaval shunts when GRWR is between 0.6% and 1% if PVP is < 20mmHg. According to the Medanta Institute protocol, PVP and GRWR are the two factors to consider for GIM. Soint el al[65] perform a hemi-porto-caval shunt (HPCS) for every graft with GRWR < 70% or with GRWR between 0.70 and 0.74% if PVP during dissection is > 18mmHg, whereas if PVP is between 16 and 18mmHg or GRWR 0.75–0.79%, they promote SAL. Importantly, the authors do not perform any GIM if PVP is ≤ 15mmHg or GRWR ≥ 0.80%.

Overall, while shunt formation represents a valuable tool in managing SFSS, its utilization demands careful assessment of patient-specific factors and close monitoring to mitigate potential complications and optimize outcomes in LDLT.

#### Retransplantation

The necessity of retransplantation (re-LT) following SFSS in LDLT represents a critical aspect of patient management, often driven by the severity of the initial syndrome, its associated complications and the failure of GIM techniques. Kurimatsu et al. [66] reported the incidence of re-LT for SFSS at 1.5% in a cohort of 256 retransplants, whereas Wong et al. [67] reported no graft loss in their study of 545 LDLT recipients, 26 of whom developed SFSS. Criteria for retransplant for SFSS are currently debated. In 2016, Pomposelli et al. reported data from the A2ALL study, which demonstrated that early allograft dysfunction (EAD), according to Olthoff criteria, occurred in 16–19% of recipients and was related to 24% of 90-day mortality compared to recipients without EAD. The risk of EAD was higher in left lobe grafts, lower GRWR, higher PVP after reperfusion, high preoperative bilirubin, older donors and higher BMI. However, 75% of recipients with EAD recovered without graft failure, thus making the decision for re-LT more complicated. In the A2ALL study, Braun et al. [68] reported a re-LT rate of 10%. In a cohort of 207 LDLT recipients, Ikegami et al. [3] demonstrated higher graft loss and higher 5-year mortality (43% Vs 94%) in recipients with severe SFSS defined as total bilirubin > 20mg/dL within a month from transplant. Notably, multivariate analysis for severe SFSS after left lobe-LDLT showed that donor age of > 48 years (*p* = 0.01), MELD ≥ 19 (*p* < 0.01), and end PVP ≥ 19 mmHg (*p* = 0.04) were the significant and independent factors for severe SFSS after left lobe-LDLT.

Recently, the ILTS-iLDLT-LTSI Consensus Conference [29] recommended considering re-LT when pharmacologic or surgical GIM have failed and the recipient persistently shows total bilirubin > 10mg/dL associated with INR > 1.6 or total bilirubin > 20mg/dL alone. According to the expert panel, the clinical status of the recipient should also be considered, particularly the presence of ascites, rising ammonia, organ dysfunction and the absence of sepsis (Level of Evidence: Low; Strength of recommendations: Moderate).

To date, further post-operative scores have been developed to predict 90-day graft-loss probability and guide the surgeon’s decision-making regarding early re-LT. The timing for re-LT in SFSS recipients is critical. Bittermann et al. [69] reported that LDLT recipients are more often critically ill at re-LT compared to recipients of deceased donor grafts. Indeed, compared to a UNOS database cohort of nearly 2900 primary transplant recipients of deceased donor livers, LDLT re-LT patients presented at retransplant with a higher MELD (29 Vs 27), intensive care unit hospitalization (52,2% Vs 39.9%; *p* < 0.001) and a higher rate of UNOS status 1A (42.6% Vs 27.3%, *p* < 0.001). Importantly, 1-year mortality and the need for a third transplant were similar between the two groups [69–71]. This result was confirmed by Braun et al. [67], who reported that the timing of re-LT did not affect post-transplant survival but increased complication rates compared to primary LDLT. Moon et al. [72] recommended using GRWR > 0.80% graft for re-LT and warned against LDLT in a recipient with a first LDLT because of the higher technical difficulty. The ILTS-iLDLT-LTSI Consensus Conference[29] recommended waiting two weeks after transplant before considering re-LT, due to the regenerative capacities of the liver, except in SFSS Grade C, which is associated with a risk of graft loss of 59–77%.

## Conclusions

Prevention and mitigation of SFSS requires careful assessment of recipient and graft characteristics. Graft/recipient matching, intraoperative hemodynamic assessment, GIM and optimization of the venous outflow are extremely valuable strategies in LDLT and have allowed the successful transplantation of ultra-small grafts (GRWR < 0.7).
